# Bioactivities and Mode of Actions of Dibutyl Phthalates and Nocardamine from *Streptomyces* sp. H11809

**DOI:** 10.3390/molecules27072292

**Published:** 2022-03-31

**Authors:** Fauze Mahmud, Ngit Shin Lai, Siew Eng How, Jualang Azlan Gansau, Khairul Mohd Fadzli Mustaffa, Chiuan Herng Leow, Hasnah Osman, Hasidah Mohd Sidek, Noor Embi, Ping-Chin Lee

**Affiliations:** 1 Institute for Research in Molecular Medicine, Universiti Sains Malaysia, Gelugor 11800, Malaysia; fauzem@ums.edu.my (F.M.); khairulmf@usm.my (K.M.F.M.); herng.leow@usm.my (C.H.L.); 2 Faculty of Science and Natural Resources, Universiti Malaysia Sabah, Kota Kinabalu 88400, Malaysia; sehow@ums.edu.my (S.E.H.); azlanjg@ums.edu.my (J.A.G.); 3 School of Chemical Sciences, Universiti Sains Malaysia, Gelugor 11800, Malaysia; ohasnah@usm.my; 4 Faculty of Science and Technology, Universiti Kebangsaan Malaysia, Bangi 43600, Malaysia; hasidah@pkrisc.cc.ukm.my (H.M.S.); noormb@pkrisc.cc.ukm.my (N.E.); 5 Biotechnology Research Institute, Universiti Malaysia Sabah, Kota Kinabalu 88400, Malaysia

**Keywords:** GSK-3β inhibitors, mixed inhibition, antimalarial, iron-chelating, dibutyl phthalate, nocardamine

## Abstract

Dibutyl phthalate (DBP) produced by *Streptomyces* sp. H11809 exerted inhibitory activity against human GSK-3β (*Hs* GSK-3β) and *Plasmodium* *falciparum* 3D7 (*Pf* 3D7) malaria parasites. The current study aimed to determine DBP’s plausible mode of action against *Hs* GSK-3β and *Pf* 3D7. Molecular docking analysis indicated that DBP has a higher binding affinity to the substrate-binding site (pocket 2; −6.9 kcal/mol) than the ATP-binding site (pocket 1; −6.1 kcal/mol) of *Hs* GSK-3β. It was suggested that the esters of DBP play a pivotal role in the inhibition of *Hs* GSK-3β through the formation of hydrogen bonds with Arg96/Glu97 amino acid residues in pocket 2. Subsequently, an in vitro *Hs* GSK-3β enzymatic assay revealed that DBP inhibits the activity of *Hs* GSK-3β via mixed inhibition inhibitory mechanisms, with a moderate IC_50_ of 2.0 µM. Furthermore, the decrease in K_m_ value with an increasing DBP concentration suggested that DBP favors binding on free *Hs* GSK-3β over its substrate-bound state. However, the antimalarial mode of action of DBP remains unknown since the generation of a *Pf* 3D7 DBP-resistant clone was not successful. Thus, the molecular target of DBP might be indispensable for *Pf* survival. We also identified nocardamine as another active compound from *Streptomyces* sp. H11809 chloroform extract. It showed potent antimalarial activity with an IC_50_ of 1.5 μM, which is ~10-fold more potent than DBP, but with no effect on *Hs* GSK-3β. The addition of ≥12.5 µM ferric ions into the *Pf* culture reduced nocardamine antimalarial activity by 90% under in vitro settings. Hence, the iron-chelating ability of nocardamine was shown to starve the parasites from their iron source, eventually inhibiting their growth.

## 1. Introduction

Actinomycetes are the leading source of bioactive compounds with numerous activities, including anticancer and antibiotic [1,2]. Malaysian rainforests are well known for their rich biodiversity, including that of soil microorganisms. We previously reported the isolation of actinomycetes strains from different forest types in Borneo, Malaysia, to identify inhibitors against proteins involved in eukaryotic cell signaling pathways, including glycogen synthase kinase-3β (*Hs* GSK-3β) [3,4,5]. GSK-3β is a serine-threonine protein kinase that regulates cell processes such as cell growth, proliferation, and survival [6]. The overexpression of GSK-3β is implicated in many diseases such as cancer, neurodegenerative diseases, and diabetes [7,8]. Hence, a GSK-3β inhibitor is an attractive target in drug screening, development, and design [9,10,11]. Due to accumulated knowledge on human GSK-3, the protozoan parasite GSK-3 was also indicated as a druggable target for treating infectious diseases, including malaria. *Plasmodium falciparum* GSK-3 (*Pf* GSK-3) is essential for *Plasmodium* survival during the erythrocytes cycle. Structurally, *Pf* GSK-3 is 75% similar to *Hs* GSK-3β [12]. A high-throughput screening of *Hs* GSK-3β inhibitors managed to identify thieno[2,3-*b*]pyridines scaffolds as selective derivatives towards *Pf* GSK-3 [13].

Our previous screening identified dibutyl phthalates (DBP) from *Streptomyces* sp. H11809 to have inhibitory activity against *Hs* GSK-3β (in vivo yeast-based assay). DBP also showed antimalarial activities against *Plasmodium falciparum* 3D7 (*Pf* 3D7) with no cytotoxic effect on Chang liver cells [14]. Although a drug’s mode of action (MoA) is not required for Food and Drug Administration (FDA) drug approval [15], knowledge of a drug’s MoA is helpful to prevent late-stage drug trial failure. Hence, this study reports the MoA of DBP against recombinant *Hs* GSK-3β activity and its possible molecular target on *Plasmodium* via resistant clone generation. In addition, nocardamine was identified as another active compound from H11809 crude extract that showed moderate antimalarial activity but no activity against *Hs* GSK-3β.

## 2. Results

### 2.1. Characterization and Classification of H11809

H11809 requires ten days for sporulation on oatmeal agar (OA) (Table S1). The aerial mycelia are light brown with no soluble pigmentation. Scanning electron microscopy revealed that the aerial mycelia of H11809 had long chains of spores with a smooth surface (Figure S1). The 16s rRNA gene sequence of H11809 showed 99% similarity to the various corresponding sequences of *Streptomyces* sp. such as *Streptomyces* sp. SllA 2050 (accession number FJ492849.1), *Streptomyces* sp. Av25_4 (accession number FJ490534.1), and *Streptomyces* sp. WW4-1s (accession number KJ143657.1) (Figure S2). H11809 sequence was deposited in the NCBI database and was identified as *Streptomyces* sp. H11809 with accession number MN602474.

### 2.2. Bioassay-Guided Fractionation and Compound Identification

H11809 crude and chloroform extracts inhibited the yeast growth at both 28 °C and 37 °C but with a larger zone of inhibition at 37 °C, indicating non-selective *Hs* GSK-3β inhibition. The H11809 chloroform extract was fractionated using column chromatography to collect 60 fractions. These fractions were pooled into nine fractions and tested against *Hs* GSK-3β yeast-based assay. Fraction 8 (H11809-CCF8) showed the most significant selective *Hs* GSK-3β inhibitory activity because the zone of inhibition was observed (only at 37 °C) (Table 1).

**Figure 1:**
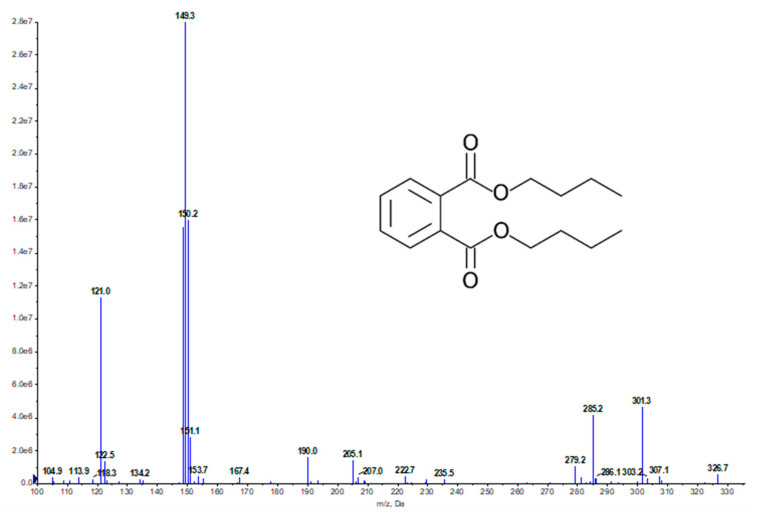
UPLC-MS analysis of H11809-CCF8. Selected column chromatography fractions of H11809 chloroform extracts were tested against *Pf* 3D7 for antimalarial activity (H11809-CCF5 to -CCF9). H11809-CCF8 (DBP was identified from this fraction) only showed antimalarial activity at high concentrations (500 µg/mL). Meanwhile, H11809-CCF9 exerted more significant activity, by which the antimalarial activity was still observed at 5 µg/mL (Table 2).

H11809-CCF8 was further analyzed using UPLC-MS. The presence of DBP was identified at a retention time (RT) of 3.7 min and a base peak at 149 *m*/*z*, a typical base peak fragmentation pattern of phthalate esters with an alkyl side chain (Figure 1) [16], and 279.2 *m*/*z* [M + H]^+^. The DNP database search identified the peak as dibutyl phthalate with the molecular formula of C_16_H_22_O_4_ and 278.3 *m*/*z*. H11809-CCF8/dibutyl phthalates (DBP): colorless oil; ^1^H-NMR (500 MHz, CDCl_3_) spectra of H11809-CCF8 showed peaks at δ 7.71 (td, J = 6.6, 3.4 Hz, 1H), 7.54–7.51 (m, 1H), 4.30 (t, J = 6.9 Hz, 4H), 1.74–1.69 (m, 4H), 1.59–1.40 (m, 4H), 0.96 (t, J = 7.4 Hz, 6H). Meanwhile, the ^13^C-NMR (126 MHz, CDCl_3_) spectra showed peaks at δ 167.9, 132.5, 131.1, 129.0, 65.8, 30.8, 19.4, 13.9 (Figure S3). The acquired data were compared with the reported NMR of dibutyl phthalates and showed similar signals for both ^1^H- and ^13^C-NMR [17] (Table S2). Furthermore, both commercial DBP and H11809-CCF8 had similar RT (Figure S4), UV maxima, and mass, confirming DBP as the active compound in H11809-CCRF8.

**Table 1:** The inhibitory activity of different fractionation stages of H11809. Column chromatography fraction of H11809-chloroform extract; H11809-CCF8 showed the most significant GSK-3β inhibitory activity.

Sample	Inhibition Zone, mm (±SD)		Remarks
	28 °C	37 °C	
H11809-crude extract	12.67 ± 0.58	15.0 ± 0.0	Non-selective inhibition
H11809-chloroform extract	7.0 ± 0.0	8.0 ± 0.0	Non-selective inhibition
H11809-CCF1	0.0 ± 0.00	0.0 ± 0.00	No activity
H11809-CCF2	0.0 ± 0.00	0.0 ± 0.00	No activity
H11809-CCF3	0.0 ± 0.00	0.0 ± 0.00	No activity
H11809-CCF4	8.0 ± 0.0	8.0 ± 0.58	Non-selective inhibition
H11809-CCF5	18.0 ± 0.58	7.0 ± 0.58	Cytotoxic
H11809-CCF6	10.0 ± 0.58	10.0 ± 0.0	Cytotoxic
H11809-CCF7	8.0 ± 0.0	10.0 ± 0.0	Non-selective inhibition
H11809-CCF8	0.0 ± 0.0	10.0 ± 0.0	Selective inhibition
H11809-CCF9	0.0 ± 0.00	0.0 ± 0.0	No activity
*Streptomyces* H7667 ^1^	13.0 ± 0.0	14.0 ± 0.0	Non-selective inhibition

“^1^” is the crude extract of *Streptomyces* H7667, a strain that produces few GSK-3β inhibitors [18].

**Table 2:** Antimalarial activity of selected H11809-chloroform extract column chromatography fractions.

Tested Conc. µg/mL	H11809-CCF5	H11809-CCF6	H11809-CCF7	H11809-CCF8	H11809-CCF9
500	+++	++	+++	+++	+++
50	++	+	-	-	+++
5	-	-	-	-	++
0.5	-	-	-	-	-

Note: +++: >80% *Pf* clearance, ++: >50% *Pf* clearance, +: >20% *Pf* clearance, -: no activity.

**Figure 2:**
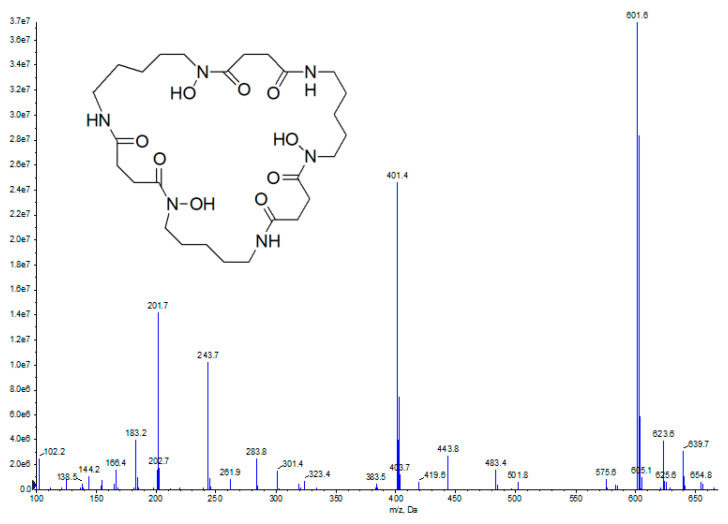
UPLC-MS analysis of H11809-CCF9.

UPLC-MS analysis of H11809-CCF9 revealed a major peak with an RT of 1.7 min (no DBP peak at RT 3.7 min), indicating the presence of another active compound with antimalarial activity but no activity against *Hs* GSK-3β. MS analysis revealed a compound with 601.6 *m*/*z* [M + H]^+^ as the parent signal. Based on the DNP database search, this compound was identified as nocardamine with the molecular formula of C_27_H_48_N_6_O_9_ and 600.7 *m*/*z*. Due to the small amount of nocardamine produced by *Streptomyces* sp. H11809 (<0.5 mg), ^1^H-NMR analysis of H11809-CCF9 was unsuccessful. However, nocardamine are known to produce a distinctive MS signal pattern at 601, 401, and 201 *m*/*z* [M + H]^+^ (corresponding to the fragment ion peaks of three repeating units of N-hydroxy-N’-succinylcadaverine) [19,20], that was also observed in the mass spectrum of H11809-CCF9 (Figure 2). Moreover, commercial nocardamine and active compounds in H11809-CCF9 had similar RT (Figure S5), UV maxima, and mass, confirming nocardamine as the active compound’s identity in H11809-CCF9. Commercial nocardamine was then tested at 16-point dilutions against *Pf* 3D7 culture. The IC_50_ of nocardamine *Pf* 3D7 was 1.5 M (moderate antimalarial activity) (Figure S6).

### 2.3. DBP Inhibits Hs GSK-3β Substrate Binding Site via Mixed Inhibition

**Figure 3:**
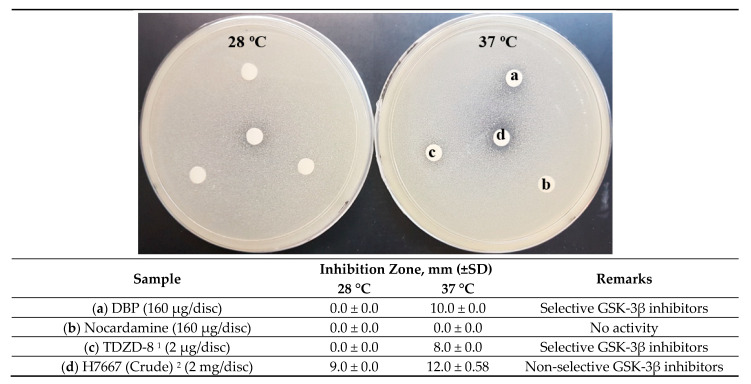
A yeast-based assay to evaluate the inhibitory activity of DBP (a) and nocardamine (b) against *Hs* GSK-3β, at 160 μg/disc. “^1^” is a selective *Hs* GSK-3β inhibitor [24]. “^2^” is the extract of *Streptomyces* sp. H7667, a strain that produces few GSK-3β inhibitors [18].

The yeast-based assay applied in this study was more sensitive towards non-ATP-competitive inhibitors such as 4-Benzyl-2-methyl-1,2,4-thiadiazolidine-3,5-dione (TDZD-8). Some ATP-competitive inhibitors, such as staurosporine, could also be identified, although a large chemical concentration was required for a detectable inhibition zone [21]. DBP showed positive inhibition (partial growth inhibition at 37 °C, 10.0 ± 0.0 mm, and no yeast-growth defects were observed at 28 °C), indicating selective GSK-3β inhibition. In addition, TDZD-8 also exerted a similar inhibition pattern (Figure 3). Based on this result, DBP and TDZD-8 are likely to inhibit GSK-3β at both temperatures (28 and 37 °C). However, only GSK-3β inhibition at 37 °C has a fatal effect. Without GSK-3, yeast cells lose cell wall integrity [22] and become temperature sensitive. Hence, it was not able to grow at 37 °C. In contrast, yeast cells survived GSK-3β inhibition at 28 °C because a compromised cell wall has no fatal effect at a lower temperature. Hence, no inhibition zone was observed at 28 °C [23]. However, a high concentration of DBP was required for the observable inhibitory effect (160 µg/disc of samples, compared to TDZD-8 at 2 µg/disc). Meanwhile, nocardamine exerted no activity on GSK-3β.

**Figure 4:**
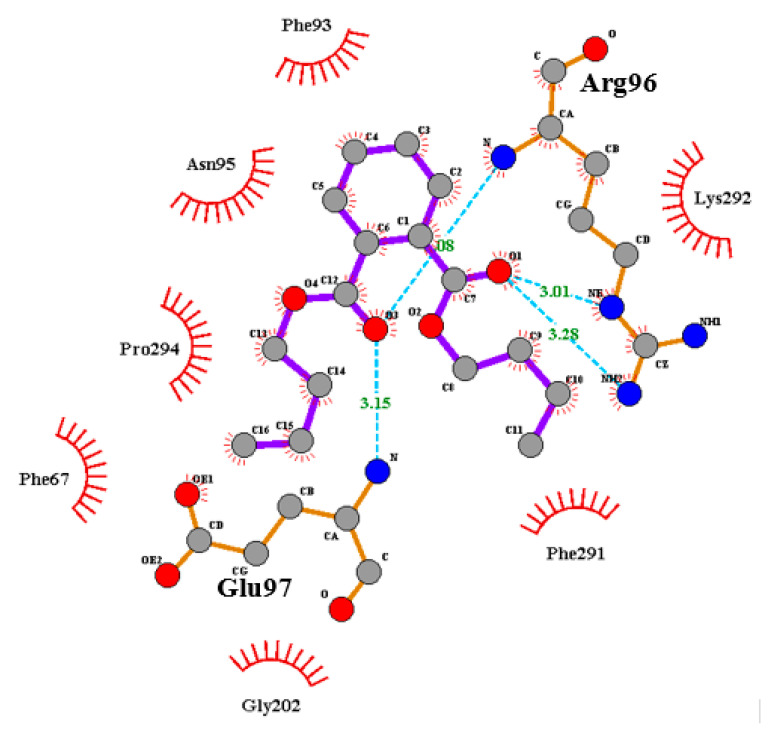
Two-dimensional Ligplot^+^ diagram of the interaction between DBP and amino acid residues on the substrate-binding site (P2) of GSK-3β. Double-bonded oxygen of ester groups in DBP form hydrogen bonds (blue dotted lines) with Arg96 and Glu97. Red, spoked arcs represent hydrophobic interactions between DBP and amino acid residues of P2. Oxygen is red, nitrogen is blue, and carbon is gray circles.

Based on the result of the yeast-based assay, a preliminary docking study of DBP interaction with *Hs* GSK-3β was performed [25,26,27]. Docking studies demonstrated weak interactions of DBP on pocket 1 (P1, ATP-binding site of GSK-3β), as only steric interactions were established. The best-fit pose for molecular interaction on P1 was chosen based on the binding affinity of DBP (−6.1 kcal/mol). In contrast, DBP established a total of four hydrogen bonds on pocket 2 (P2, substrate-binding site of GSK-3β), by which two hydrogen bonds formed with Arg96 (3.01 Å, −2.23 kcal/mol and 3.28 Å, −0.06 kcal/mol) and Glu97 (3.15 Å, −2.24 kcal/mol and 3.07 Å, −0.32 kcal/mol), respectively (Figure 4). These hydrogen bonds resulted in a higher binding affinity of DBP on P2 than P1; the best-fit pose had a −6.9 kcal/mol binding affinity. In addition, non-bonded interaction (electrostatic or Van der Waals interactions) was also established between DBP and Tyr216, which is a crucial residue that controls the activation of GSK-3β [28] (Table 3). This result indicates that compound DBP is likely to bind on the substrate-binding site, P2, instead of the ATP-binding site, P1.

**Figure 5:**
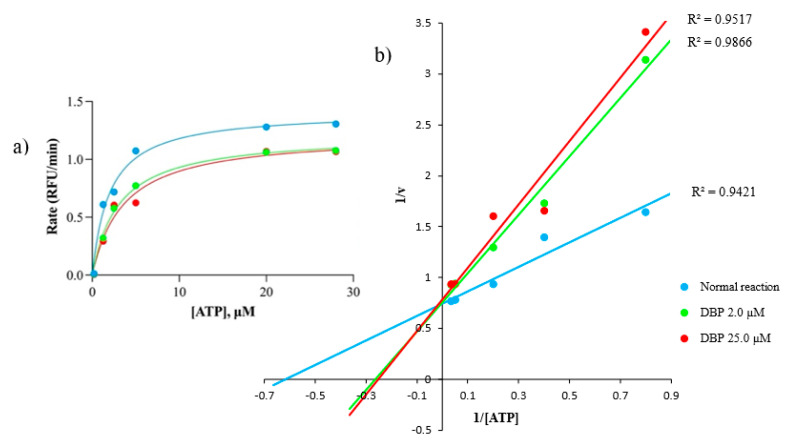
Michaelis−Menten analysis (**a**) indicates DBP increased the K_m_ but decreased the V_max_ values. For more accurate V_max_ and K_m_ value determination, the Lineweaver−Burk graph was plotted (**b**). GSK-3β inhibition by DBP is significant with *p* = 0.0017 (mixed inhibition).

Based on the yeast-based assay and in silico study, DBP was further tested against the *Hs* GSK-3β enzymatic assay. This assay measures the amount of free ATP before and after the enzymatic reactions. The in vitro enzymatic assay showed that DBP causes higher ATP accumulations after the enzymatic reaction than a normal reaction. As the concentration of DBP was increased, the K_m_ value was also increased, and conversely for the V_max_ value (Michaelis–Menten plot), indicating enzyme inhibition [29]. For accurate K_m_ and V_max_ determination (the x- and y-intercept represents −1/K_m_ and 1/v values, respectively), the Lineweaver–Burk graph was plotted [30]. It was also observed that the *Hs* GSK-3β inhibition by DBP is significant, *p* = 0.0017 (95%-confidence) (Figure 5) (Table 4). Based on K_m_ and V_max_ changes, the inhibitory mechanism exerted by DBP against *Hs* GSK-3β is concluded to be mixed inhibition (IC_50_ = 2.0 µM, a moderate inhibitor) (Figure S7). Mixed inhibition is almost similar to a non-competitive inhibitor, except its K_m_ value will also be affected by increased inhibitor concentration [31].

### 2.4. Antimalarial Mode of Action Study

#### 2.4.1. Mutant Resistance Generation against DBP

In the previous study, we reported the antimalarial activity of DBP against *Pf* 3D7 [14]. To study its antimalarial MoA, *Pf* 3D7 was exposed to an increasing concentration of DBP to generate a DBP-resistant mutant clone. However, a significant resistance mutant clone against DBP could not be developed, as no more than 2-fold resistance was observed (*Pf* 3D7 strain exposed to 40 μM DBP, the highest concentration possible). Due to insignificant resistance, further genomic sequencing was not performed. Hence, the antimalarial MoA of DBP remains unknown.

#### 2.4.2. Iron-Dependency of Nocardamine to Exert Antimalarial Activity

Nocardamine is an iron-chelating agent that binds to iron ions in cells. Based on the *Pf* 3D7 IC_50_ of nocardamine, 20 μM (>90% *Pf* clearance) was selected as a single concentration to be tested in the presence of 16-dilution points (half dilution of the previous at each point) of FeCl_3_ concentrations (the highest and lowest concentrations were 50 μM and 0.002 μM, respectively). FeCl_3_ diluents were first tested against *Pf*-infected blood cells to observe any adverse effect on the culture. The addition of FeCl_3_ posed no negative effect on *Pf* culture (each well has >95% *Pf* infection rate after 72 h incubation). Subsequently, the FeCl_3_ diluents were added to *Pf* culture with 20 μM nocardamine in each well and incubated for 72 h. It was found that ≥12.5 FeCl_3_ can reduce the antimalarial effect of 20 μM nocardamine by 90% (Table S3). The iron-dependency experiment confirmed that antimalarial activity exerted by nocardamine is iron-dependent, as an increasing concentration of Fe^3+^ saved *Pf* culture from nocardamine inhibitory activity (Figure S8).

## 3. Discussion

DBP is a phthalic acid ester characterized as a clear, colorless, oily liquid that is insoluble in water. DBP is a product of the shikimic acid pathway in microorganisms [32]. DBP from several actinomycetes strains such as *Streptomyces* sp. YYS-7 and *Streptomyces albidoflavus*, which displayed antimicrobial and fungal activities, has been reported previously [33,34]. Enzymatic assay suggests that DBP inhibits *Hs* GSK-3β via the mixed inhibition action.

Furthermore, an increase in K_m_ value indicates that DBP favors binding on free *Hs* GSK-3β over *Hs* GSK-3β-substrate complexes, which causes a lower binding affinity of GSK-3β towards its substrates, eventually lowering the V_max_ value (Figure S9). To determine the possible binding site of DBP on free *Hs* GSK-3β, we conducted a preliminary in silico study targeting two active sites on *Hs* GSK-3β: ATP-binding pocket (P1) and substrate-binding pocket (P2). Our docking study revealed that DBP might interact with critical amino acid residues on P2, mediated by double-bonded oxygen of DBP functional groups (ester). DBP can form a hydrogen bond with Arg96, which is involved in the P + 4 phosphates binding site (P-loop). The second double-bonded oxygen of DBP creates hydrogen bonding with Glu97, part of three amino acid residues (including Asp200 and Lys85) that can be targeted to enhance activity and selectivity against *Hs* GSK-3β [35].

Although hydrophobic interactions are weak, their effect cannot be neglected as the number of atoms involved significantly contributes to a specific interaction between enzyme and substrate. Interaction with amino acids residues provides essential information on its inhibitory activity and MoA. Significant non-bonded interaction of DBP on P2 is on the “hydrophobic patch”, which is located in the C-terminal lobe of GSK-3β (facing the ATP-binding site) that consists of Val214, Ile217, and Tyr216 [26]. Tyr216 is pivotal for the activation of *Hs* GSK-3β, in which the binding of DBP on P2 will likely prevent GSK-3β activation. TDZD-8, the first *Hs* GSK-3β proven non-ATP-competitive inhibitor, binds to P2 by forming hydrogen bonds with Arg96, Arg180, and Lys205. In addition, it also interacts with Try216 and Val214 through hydrophobic and π-stacking, which prevent the activation of GSK-3β [36].

A highly potent GSK-3β inhibitor is widely perceived as one of the main criteria in drug discovery. This is indicated by low IC_50_ values, such as those exerted by staurosporine (15 nM) and indirubin (22 nM) [37,38]. However, diseases associated with GSK-3 are not caused by more than 2- or 3-fold increases in regular GSK-3 activity [36]. Thus, about 50% of the inhibition rate is sufficient for effective treatment for these diseases. It is suggested that a moderate inhibitor is more desirable for a sustainable long-term treatment plan as the complete inhibition of protein kinase frequently causes adverse side effects such as deleterious effects on healthy tissues [39,40]. In this study, inhibitory activity exerted by DBP against GSK-3β is considered moderate. Thus, DBP or its derivatives can be an interesting lead for the development of GSK-3β inhibitors in treating diseases resulting from GSK-3β dysregulation in humans.

Antimalarials’ molecular targets can be discovered by creating drug-resistant parasites and comparing the genomes of the resistant clones to the wild type [41]. This method has led to the identification of numerous novel targets, including *Pf* ATP4 and *Pf* CARL [42]. In this study, the generation of a *Pf* mutant strain against DBP via continuous drug exposure was unsuccessful, suggesting DBP’s plausible target might be indispensable for *Pf* survival. Although the effect of DBP on *Pf* GSK-3 has yet to be determined, it has been reported that DBP inhibits GSK-3 activity in other species. DBP was reported to cause a higher accumulation of β-catenin in the nucleus of Zebrafish embryos, resulting in abnormal development. Accumulations of β-catenin in the nucleus may occur due to the inhibition of GSK-3β that prevents its phosphorylation and proteasomal degradation. However, the Western blot showed that DBP does not affect the Ser9 phosphorylation level of Zebrafish embryos [43]. In contrast, the inhibition of GSK-3β by DBP increases the GSK-3β Ser9 phosphorylation level in the liver of male, six-week-old ICR mice infected with *Plasmodium berghei,* by which DBP was implicated as a potential malaria treatment in vivo [14]. This contradictory finding may be caused by slight structural differences of the GSK-3β kinase domain between different species [12].

*Pf* GSK-3 was genetically and phenotypically validated as an antimalarial therapeutic target [44,45]. The physiological function of *Pf* GSK-3 is not fully understood. However, it is essential for the egression of *Plasmodium* during the erythrocytic stage [13,46]. Interestingly, *Pf* GSK-3 knock-out was also reported to be unsuccessful in *P. falciparum* via site-directed mutagenesis [46], which is corroborated by the unsuccessful mutant generation in this study. Given drug-resistance development is a major problem in the malaria endemic, a scaffold that is not prone to resistance generation by *Plasmodium* should be prioritized [47]. This evidence suggests the *Pf* GSK-3 inhibitor as a potential antimalarial target. Hence, a different strategy needs to be applied to understand the effect of DBP, and eventually to screen for more *Pf* GSK-3 inhibitors for future study.

Moreover, *Hs* GSK-3β was reported to modulate the inflammatory response activated by parasite infections, marked by a dramatic increase in cytokine production [48]. Cytokine storms are usually implicated in cerebral malaria and liver damage, which can be fatal without treatment [49,50,51]. We previously reported the dual effect exerted by DBP in *P. berghei* infected mice as antimalarial agents and in inhibiting the activity of host GSK-3β (increased Ser9 phosphorylation) [14]. In addition, we also reported quercetin (*Hs* GSK-3β inhibitor) exerted both antimalarial and host GSK-3β-mediated cytokine levels in the brain of *P. berghei*-infected mice [52]. Targeting a host enzyme that the parasite cannot mutate provides a new therapeutic insight that will prevent severe malaria and the development of resistant strains [53,54]. Dual effects exerted by GSK-3 inhibitors might help overcome and compensate the need for new antimalarial target with a longer clinical lifespan.

Meanwhile, nocardamine is a well-known iron-chelating compound [19]. Since iron is essential for *Pf*’s growth [55], it is expected that antimalarial activity exerted by nocardamine is via *Plasmodium* iron starvation. Iron is essential for human physiological and cellular processes, including the immune system, DNA replication and repair, mitochondrial function, and erythropoiesis [56]. Ferroportin (FPN), the only known iron transporter in humans, is highly abundant in red blood cells. It is vital to protect cells from toxic iron accumulation generated by hemoglobin autoxidation and malaria infection. In *Plasmodium*, iron is especially required during the erythrocytes stage, as rapid parasite proliferation requires iron as the cofactor for DNA replication enzyme [57]. Anemic patients were reported to be in a protective condition that prevented malarial infections. In addition, individuals with increased ferroportin levels acquired a protective effect against *Plasmodium.*

In contrast, *fpn* gene knock-out mice developed a malaria-susceptible phenotype. Moreover, iron supplementary therapy for anemic patients was reported to increase malaria risk, because additional iron inhibits the activity of FPN. Hence, iron levels in the blood, regulated by FPN, influence malaria infection in humans [58]. For decades, iron chelators have gained attention in antimalarial drug development, recognizing the impact of iron levels in blood with malaria infection susceptibility [59]. Siderophores are among the most potent known ferric ions binding agents that are vastly secreted by microorganisms. It is vital for iron transport across the membrane [60]. Siderophores have been reported to exert diverse bioactivities [61], including antimalarial activity [62]. The gene cluster encoding siderophore biosynthesis represents one of the main and common secondary metabolites gene clusters in actinomycetes [63]. The most commonly produced siderophore by actinomycetes is desferrioxamine (DFO), with a reported *Pf* 3D7 IC_50_ of 14 μM [64,65]. We identified nocardamine (desferrioxamine E) as the active compound against *Pf* 3D7, which has a moderate *Pf* IC_50_, and its antimalarial activity is significantly reduced (>90%) in the presence of excessive ferric ions (≥12.5 μM).

RBC-stage *Pf* parasites are more sensitive to iron chelators (at μM concentration) than mammalian cells (mM concentration). DFO has been reported to affect malaria in a stage-specific manner, especially against the late trophozoites and schizont stages [66]. On the contrary, some siderophores’ antimalarial modes of action have been previously reported to involve more complex cellular processes such as the direct inhibition of parasite ribonucleotide reductase activity and the modification of the host immune response rather than simple iron starvation [67,68]. Although >95% of the iron in the blood is in the form of the heme [69], hemoglobin is not the iron source for *Plasmodium* growth. The effect of iron deficiency in anemic patients would never induce a protective effect against malaria, since only 1% heme ion would be enough for *Plasmodium* growth [70,71]. Hence, non-heme iron is essential for *Plasmodium* growth in mature erythrocytes [72]. Furthermore, DFO has low membrane solubility [73], implying that its antimalarial effect is primarily based on its ability to bind to non-heme irons, depriving the *Plasmodium* parasite of its principal iron source, at least in vitro, as demonstrated in this study.

## 4. Materials and Methods

### 4.1. Isolation, Cultivation, Microscopic Characterization, and Molecular Identification of H11809

H11809 was isolated from a soil sample collected at 15 cm depth during the expedition to Imbak Valley, Sabah, Malaysia, in 2000. This strain was isolated using modified humic acid and purified on oatmeal agar [3]. Aerial mycelia from a 2-week-old culture were observed under a scanning electron microscope (SEM) for microscopic characterization. The spores were first coated in gold using the Emitech K550X sputter coater. Gold-coated spores were observed using Carl Zeiss EVO MA10 at 800× to 3k× magnifications. For molecular identification, a pure culture of H11809 was grown in mannitol–peptone broth for 34–48 h. DNA extraction and 16S rRNAs polymerase chain reaction (PCR) was performed as described previously [62]. The PCR product was purified using a QIAquick PCR Purification Kit (Qiagen, Valencia, Spain) and subjected to Sanger sequencing. Basic local alignment search tool (BLAST) analysis was performed using the National Centre for Biotechnology Information (NCBI) database to identify near genera.

### 4.2. Secondary Metabolites Production from H11809

A single colony of H11809 culture was inoculated into 10 mL of mannitol–peptone broth as a seed medium (in 125 mL flask) and incubated at 28 °C, 220 rpm for five days. The culture conditions were optimized in mannitol–peptone broth and grown for secondary metabolite production. The metabolites were extracted using acetone to obtain crude extract. The crude extract was centrifuged at 4000 rpm for 10 min to remove the mycelium. The sample was then filtered with Whatman Paper No. 1 to remove cell debris. The crude extract was freeze-dried to eliminate acetone and water.

### 4.3. Bioassay-Guided Fractionation of DBP and Nocardamine

#### 4.3.1. Yeast-Based Assay Expressing *Hs* GSK-3β

Bioassay-guided fractionation was performed using a yeast-based assay as a preliminary assay for GSK-3β inhibition activities. The yeast-based assay was developed by deleting four GSK-3β homologs genes in yeast (*MCK1*, *MDS1*, *MRK1*, and *YOL128c*). In yeast, the GSK-3 homolog is important for processes such as cell division and cell wall integrity [22]. Hence, the GSK-3-null strain is a temperature-sensitive yeast that is unable to grow at 37 °C due to a compromised cell wall at a higher temperature. The insertion and expression of *Hs* GSK-3β suppressed this fatal effect. For screening, the yeast was grown at 28 °C (no adverse effect on yeast) and 37 °C (damaging the cell wall in the absence of GSK-3) for five days. Paper discs were pipetted with 20 µL (2 mg) samples [23]. TDZD-8, a known non-ATP competitive inhibitor of GSK-3β, was used as a positive control (2 μg/disc).

A total of four outcomes can be interpreted based on the yeast inhibition pattern: (i) in the presence of selective GSK-3β inhibitor (indicated by a zone of inhibition only at 37 °C. This is because GSK-3β inhibition will cause yeast cells to lose their cell wall integrity and break at high temperatures. Meanwhile, the selective inhibition of GSK-3β at 28 °C will not affect yeast growth as a lower temperature will not cause yeast cells to break despite disrupted cell wall), (ii) non-selective GSK-3β inhibitor (inhibition on both temperatures, but larger or clearer at 37 °C. Since GSK-3 inhibition does not cause growth defects at 28 °C, if a zone of inhibition is observed at this temperature, it indicates that the tested compound is inhibiting other cell components), (iii) toxic activity (inhibition at both temperatures with an equal diameter), and (iv) no activity (no inhibition zone at both temperatures) [18,21]. The dried crude extract of H11809 was re-dissolved in sterile distilled water and partitioned based on polarity using liquid–liquid extraction to obtain hexane, chloroform, and butanol extracts. The H11809 chloroform extract, the most potent extract against GSK-3β, was further fractionated using column chromatography using the isocratic mode with 100% ethyl acetate. A total of 60 fractions were collected and pooled into nine fractions, based on the retention factor on thin-layer chromatography. Active fractions were then analyzed using UPLC-MS.

UPLC analysis was performed using an ACQUITY UPLC^®^ H-Class System (Waters, Milford, MA, USA) equipped with a quaternary solvent delivery manager pump, an auto-sampler manager-FTN, a column manager, and a photodiode array detector (connected to Empower 2 software). The detection wavelength was set at 190–600 nm with a sample injection volume of 5.0 μL. A Waters Xterra^®^ MS C18 column (2.1 × 150 mm, 5 μm) was used for separation at 40 °C. The binary gradient elution system consisted of solvent A (0.05% formic acid aqueous solution) and solvent B (acetonitrile) at a 0.5 mL/min flow rate. The solvent B gradient program was as follows (time, % of solvent B): 0.00 min, 5%; 4.00 min, 100%; 8.00 min, 100%; and 8.05 min, 5%. The entire run was 10 min: 8 min for the analysis and two additional minutes for re-equilibration.

The mass was analyzed using the API 3200^TM^ LC/MS/MS system. The mass spectra were acquired in both positive and negative mode by which the ions were scanned from *m*/*z* 100 to *m*/*z* 1500 amu; curtain gas (CUR): 15.0 psi; ion spray voltage (IS): −4500 V and 4500 V (in negative and positive ionization mode, respectively); source temperature 400 °C; ion source gas 1 (GS1) (nebulizer gas): 40 psi; ion source gas 2 (GS2) (auxiliary gas): 50 psi; delustering potential (DP): 45 V; and entrance potential (EP): 10 V.

To confirm the identity of the active compounds, ^1^H- and ^13^C-NMR were acquired using a JOEL JNM-ECA-500 spectrometer at 500 and 125 MHz, respectively. CDCl_3_ (δH 7.26 and δC 77.2) was used as a solvent and internal standard. The acquired data were then compared with reported data. In addition, commercially available DBP (Acros Organics, Geel, Belgium) and nocardamine (abcam) were purchased and analyzed using UPLC-MS to compare its RT with the active compounds identified in this study.

#### 4.3.2. Antimalarial Assay against *Plasmodium Falciparum* 3D7 (*Pf* 3D7)

The antimalarial activity of *Streptomyces* sp. H11809 was evaluated using an in vitro phenotypic assay against *Pf* 3D7 (a chloroquine-sensitive strain). The assay was performed as previously reported [74]. Briefly, the assay was performed on a 96-flat-bottom well, by which each well contained 100 µL of *Pf* culture (*Pf* infection rate and RBC concentration were adjusted to 0.3% and 2%, respectively). For crude extracts/fractions, the samples were dissolved in 100 µL of DMSO to a 100 mg/mL stock solution. Meanwhile, 50 µM sample stock solutions were prepared in 100 uL of DMSO for pure compounds. The stock solution was then diluted up to 24-dilution points using DMSO (each well was ½ concentration of the previous). For screening, 0.5 µL of the sample was pipetted into a 96-flat-bottom well with *Pf*-infected RBC. The screening plate was incubated for 72 h at 37 °C. After incubation, the plate was frozen at −80 °C for 12–24 h and thawed to break the cells. The parasite lactate dehydrogenase (pLDH) assay was performed to determine the *Pf* clearance exerted by the tested sample.

### 4.4. Mode of Action Study

#### 4.4.1. In Vitro Enzymatic Assay against *Hs* GSK-3β and Its Preliminary In Silico Analysis

DBP and nocardamine were tested against mammalian GSK-3β using an in vivo yeast-based assay. Each compound was tested at 160 μg/disc. Only DBP was shown to inhibit mammalian GSK-3β in vivo, and it was further tested against *Hs* GSK-3β. The assay was performed using a Kinase-Glo Plus Luminescent Kinase Assay (Promega, Madison, WI, USA) in a solid white 96-well plate as recommended by the manufacturer. In brief, the assay was performed at an optimized concentration of a pre-phosphorylated GSK-3β substrate (H-Leu-Ser-Glu-Thr-Lys-Pro-Ala-Val-OH) (Santa Cruz) (2 µM) and GSK-3β enzyme (20 ng), with various concentrations of ATP (0.2, 1.25, 2.5, 5.0, 20.0 and 30.0 μM). DBP was tested at 0.0 (normal reaction), 2.0, and 25.0 μM. The relative fluorescence unit (RFU) was measured after 10 min of reaction using Fluoroskan Ascent FL (Thermo Scientific, Waltham, MA, USA). To determine the inhibitory mechanisms of DBP, the rate of reaction, v (RFU/min), of the normal reaction and inhibited response was measured. The IC_50_ value of DBP against *Hs* GSK-3β was also determined. Graphs were plotted using GraphPad Prism software.

The ligand-bound structure of human GSK-3β (*Hs* GSK-3β) used in this study (Protein Data Bank (PDB) file 1Q5K, resolution 1.94 Å) was deposited by [27]. The ligand-binding sites were determined according to analysis performed by [25,26] in which pocket 1 (P1) (ATP-binding site; Tyr134, Asp133, Lys85, Val35, Cys199, Asp200, Leu188, Thr138, and Asn186) and pocket 2 (P2) (substrate-binding site; Gln89, Lys94, Asn95, Phe93, Phe67, Glu97, Arg96, Tyr216, Lys205, and Arg180) were chosen. Docking was performed using Mcule, a web-based docking application based on Autodock Vina [75,76]. GSK-3β structure was prepared with the default parameters, which resulted in the removal of water molecules. Ligand structure was generated from SMILES strings of Mcule. Visualization was performed using Molegro and LigPlot^+^ software [77].

#### 4.4.2. Antimalarial Mode of Action Study

*Pf* 3D7 culture was exposed to an increasing concentration of DBP, starting from its *Pf* IC_50_ until the maximum concentration that allowed *Pf* growth to generate a mutant *Pf* clone. To investigate if a DBP-resistant clone could be produced, the activity of DBP on WT *Pf* 3D7 was compared to that of exposed *Pf* 3D7.

To confirm whether the iron-chelating action of nocardamine is responsible for its antimalarial activity, the effect of nocardamine on *Pf*-infected RBC in the presence of iron supplement was evaluated. The effect of iron addition was first evaluated on non-infected and *Pf*-infected RBC in the absence of nocardamine to determine the suitable range of Fe^3+^ concentrations. Fe^3+^ above 50 μM caused an observable cytotoxic effect on blood cells. Based on this cut-off point, the effect of 20 μM nocardamine (the IC_90_) antimalarial activity was determined in the presence of 0.002 μM to 50 μM norcardamine.

## 5. Conclusions

Bioactive compounds are abundant in actinomycetes. However, many of these compounds failed the late stage of drug trials due to a poor understanding of their MoA. A drug’s MoA is vital in assessing its safety and dose. Our research demonstrated DBP inhibits *Hs* GSK-3β via mixed inhibition. DBP binds on substrate-binding sites and prefers free *Hs* GSK-3β over the substrate-bound *Hs* GSK-3β. DBP is an intriguing molecule to investigate because it is a non-ATP-competitive GSK-3 inhibitor with a more selective MoA. This finding, however, raises important questions about whether DBP antimalarial effectiveness is linked to the suppression of *Pf* GSK-3, a critical enzyme for *Plasmodium* survival throughout its life cycle. A different approach needs to be applied to study its antimalarial MoA. Furthermore, we showed that nocardamine is linked to non-heme iron to starve *Plasmodium* growth as its primary MoA, which is the principal iron supply for *Plasmodium* growth in an in vitro setting.

## Figures and Tables

**Table 3:** A summary of DBP-interacted amino acid residues on P1 and P2 [25] as analyzed by Molegro docking software. Enclosed and non-enclosed residues are hydrogen bond formations and hydrophobic interactions between GSK-3β with DBP, respectively.

P1 (ATP-Binding Site)	P2 (Substrate Binding Site)
Ala83, Arg141, Asp133, Asp200, Cys199, Gln185, Glu137, Gly63, Ile62, Leu132, Leu188, Lys85, Pro136, Thr138, Tyr134, Val70, Val110, Val135 Binding affinity = −6.1 kcal/mol	Ala204, (Arg96), Arg180, Asn95, Gln89, (Glu97), Gly202, Ile217, Lys85, Lys94, Phe67, Phe93, Ser203, Tyr216, Val87, Val214 Binding affinity = −6.9 kcal/mol

**Table 4:** The evaluation of V_max_ and K_m_ from Lineweaver–Burk plot. The changes in V_max_ and K_m_ patterns between normal and inhibited reactions were used to determine the inhibitory mechanisms of DBP against GSK-3β.

Rate	Normal Reaction	DBP, μM		Remarks
		2.0	25.0	
V_max_, RFU/min	1.4	1.3	1.2	Decreasing V_max_ and increasing K_m_ as DBP increased, indicating mixed inhibition
K_m_, µM	1.6	3.8	4.0	

## Data Availability

Not applicable.

## Supplementary Materials

The following supporting information can be downloaded at: https://www.mdpi.com/article/10.3390/molecules27072292/s1, Table S1: Optimization of secondary metabolites production against *Hs* GSK-3β from *Streptomyces* sp. H11809; Table S2: The comparison of ^1^H- and ^13^C-NMR of H11809-CCF8 and reported NMR analysis of DBP [17]; Table S3: The effect of Fe^3+^ addition on antimalarial activity of nocardamine. Values above the bold lines indicated reduced >90% of nocardamine antimalarial effects as ≥12.5 μM ferric ions were added into the culture; Figure S1: Morphology of *Streptomyces* sp. H11809. (**a**) *Streptomyces* sp. H11809 single colony grown for 14 days on OA. (**b**) Scanning electron micrograph showing spore chain morphology of *Streptomyces* sp. H11809 at 3.00kx magnification; Figure S2: Phylogenetic tree of strain H11809. This strain is closely related to *Streptomyces* sp. such as *Streptomyces* sp. SIIA 2050 and *Streptomyces* sp. cr36; Figure S3: The ^1^H- and ^13^C-NMR spectra of H11809-CCF8; Figure S4: UPLC chromatogram of H11809-CCF8 (green) and commercial DBP (red), 5 µL (10 µg/mL) of working solution injected into UPLC, and the chromatogram was viewed at 254 nm. Both compounds have similar RT (~3.7 min), hence confirming the presence of DBP in H11809-CCF8; Figure S5: UPLC chromatogram of H11809-CCF9 (green) and commercial nocardamine (red), 5 µL (10 µg/mL) of working solution injected into UPLC, and the chromatogram was viewed at 200 nm. Both compounds have similar RT (~1.6 min), confirming the presence of nocardamine in H11809-CCF9; Figure S6: *Pf* 3D7 IC_50_ determination of nocardamine (1.5 µM). The IC_50_ graph was plotted with GraphPad prism; Figure S7: The IC_50_ determination of DBP against *Hs* GSK-3β. The IC_50_ of DBP against *Hs* GSK-3β was 2.0 µM, a moderate *Hs* GSK-3β inhibitor. The IC_50_ graph was plotted with GraphPad prism; Figure S8: Suggested antimalarial MoA of nocardamine. The addition of ≥12.5 μM Fe^3+^ reduced the efficiency of nocardamine by 90% against *Pf* 3D7, indicating *Pf* iron starvation as the antimalarial MoA of nocardamine; Figure S9: Suggested *Hs* GSK-3β inhibition MoA by DBP (I), postulated based on K_m_ and V_max_ changes. DBP was indicated to inhibit *Hs* GSK-3β via a mixed inhibition (a combination of competitive and uncompetitive inhibitions patterns). However, the increase in K_m_ values indicates that DBP favors/higher affinity to bind on free *Hs* GSK-3β/enzyme (E) than enzyme–substrate complexes (ES). E = enzyme, S = substrate, I = inhibitor, ES = rnzyme–substrate complex, EI = enzyme–inhibitor complex, ESI = enzyme–substrate–inhibitor complex. Created with BioRender.com.

## Sample Availability

Samples of the compounds are available from the authors.
