# Supplementary material for: Bioactivities and Mode of Actions of Dibutyl Phthalates and Nocardamine from Streptomyces sp. H11809
Source: Molecules. 2022 Mar 31;27(7):2292. doi: 10.3390/molecules27072292 (PMC9000801; doi:10.3390/molecules27072292)
Supplement: Supplementary file 1 [file molecules-27-02292-s001.zip › molecules-1653324-supplementary.pdf]

Supplementary Materials

Table S1. Optimization of secondary metabolites production against *Hs*GSK-3β from *Streptomyces* sp. H11809

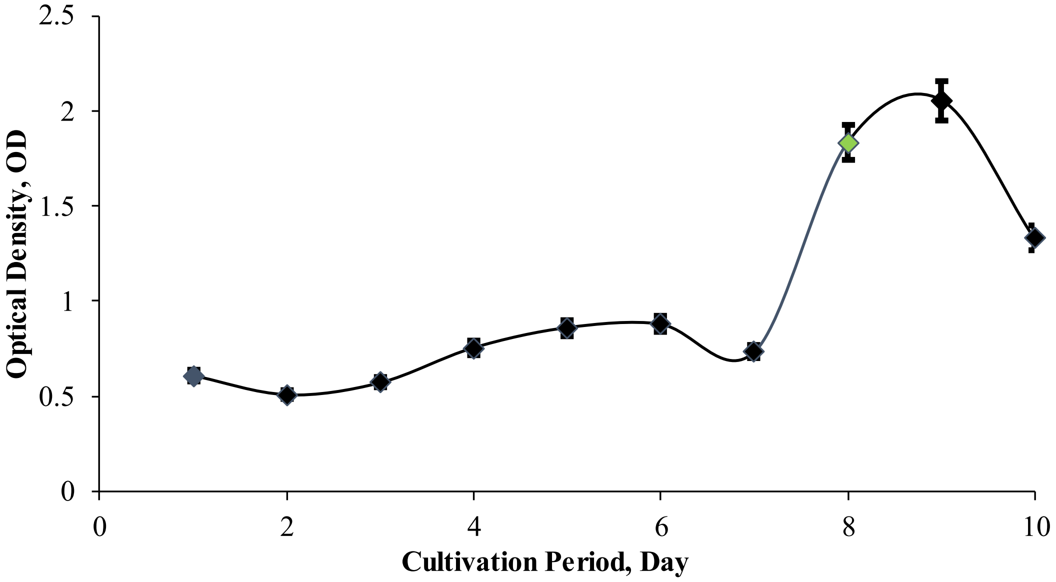

The incubation period, day	Optical density, A	Inhibition of *Hs*GSK-3β, mm	
		25 °C	37 °C
1	0.6±0.007	0	0
2	0.5±0.003	0	0
3	0.6±0.007	0	0
4	0.80±0.003	0	0
5	0.9±0.019	8.0 ± 0.0	8.0 ± 0.0
6	0.9±0.009	9.0 ± 0.0	11.7 ± 0.58
7	0.7±0.005	11.0 ± 0.0	12.3 ± 0.58
8	1.8±0.005	12.7 ± 0.58	16.0 ± 0.0
9	2.1±0.0542	11.3 ± 0.58	14.3 ± 0.58

Table S2 The comparison of ^{1}H- and ^{13}C-NMR of H11809-CCF8 and reported NMR analysis of DBP.

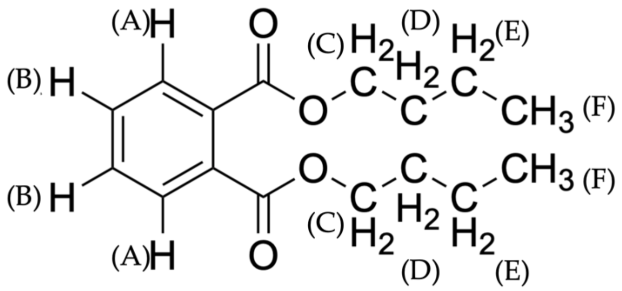

Assignment	Shift (ppm)	
	^{1}H-NMR of H11809-CCF8	Reported ^{1}H-NMR of DBP [17]
A	7.71	7.70
B	7.54-7.51	7.53
C	4.30	4.30
D	1.74-1.69	1,70
E	1.44	1.44
F	0.96	0.97

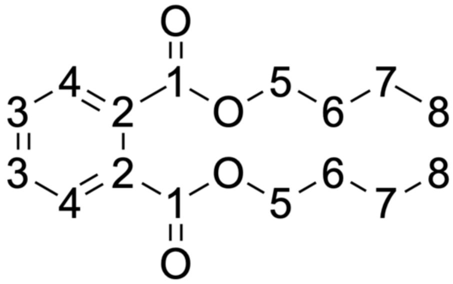

Assignment	Shift (ppm)	
	^{13}C -NMR of H11809-CCF8	Reported ^{13}C -NMR of DBP [17]
1	167.9	167.7
2	132.5	132.4
3	131.1	131.0
4	129.0	128.9
5	65.8	65.52
6	30.8	30.6
7	19.4	19.2
8	13.9	13.7

Table S3. The effect of Fe^{3+} addition on antimalarial activity of nocardamine. Values above the bold lines indicated reduced >90% of nocardamine antimalarial effects as ≥12.5 μM ferric ions were added into the culture.

**[Fe^{3+}], μM (16-dilution points)**	**IR (%) of *Pf* 3D7 + $FeCl_3$**	**IR (%) of *Pf* 3D7 + 20 μM Nocardamine + $FeCl_3$**
50	89.6	97.8
25	89.8	93.9
12.5	93.0	100.0
6.25	89.9	10.2
3.125	90.4	7.7
1.563	90.4	8.2
0.781	89.3	8.8
0.391	98.7	9.0
0.195	96.3	8.8
0.098	91.9	8.1
0.049	90.4	7.3
0.024	90.1	7.5
0.012	93.2	7.1
0.006	95.1	7.1
0.003	94.8	8.2
0.002	100.0	9.4

Note:- *Pf* 3D7 IC_{50} of nocardamine is 1.5 μM. IR = Infection rate

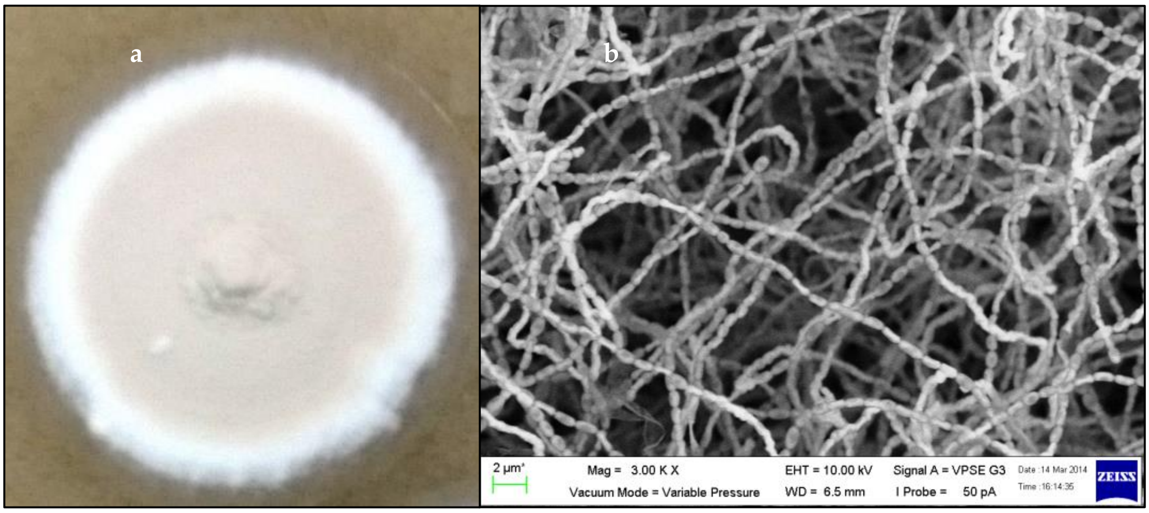

Figure S1. Morphology of *Streptomyces* sp. H11809. (a) *Streptomyces* sp. H11809 single colony grown for 14 days on OA. (b) Scanning electron micrograph showing spore chain morphology.

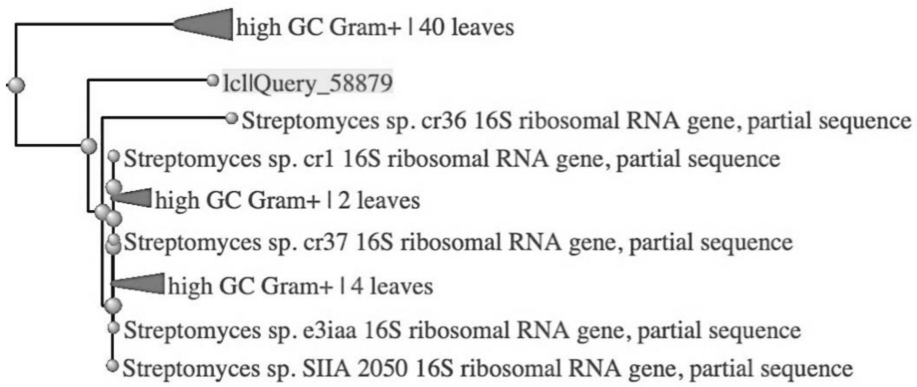

Figure S2. Phylogenetic tree of strain H11809. This strain is closely related to *Streptomyces* sp. such as *Streptomyces* sp. SIIA 2050 and *Streptomyces* sp. cr36.

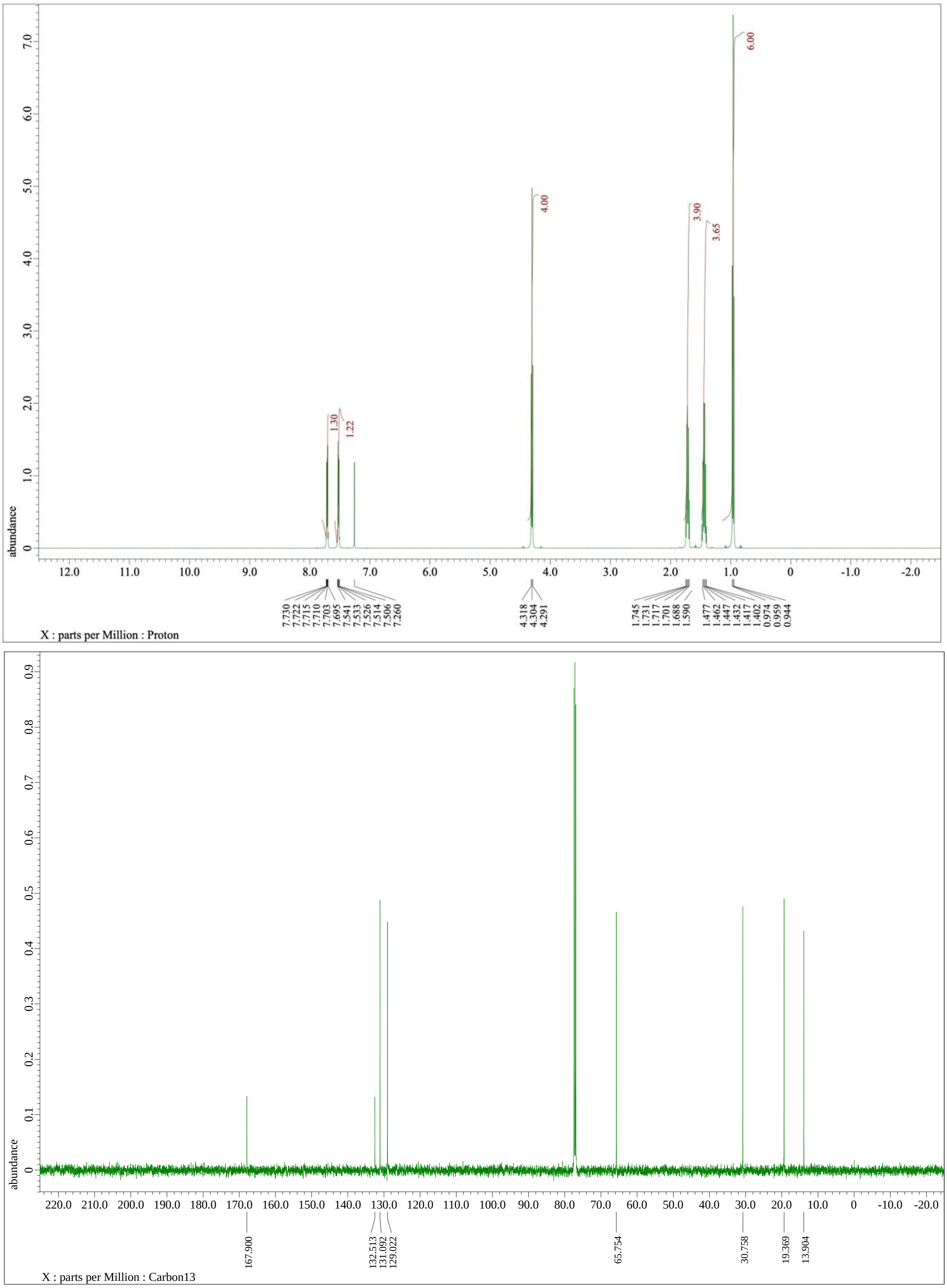

Figure S3. ^{1}H- and ^{13}C-NMR spectrum of H11809-CCF8.

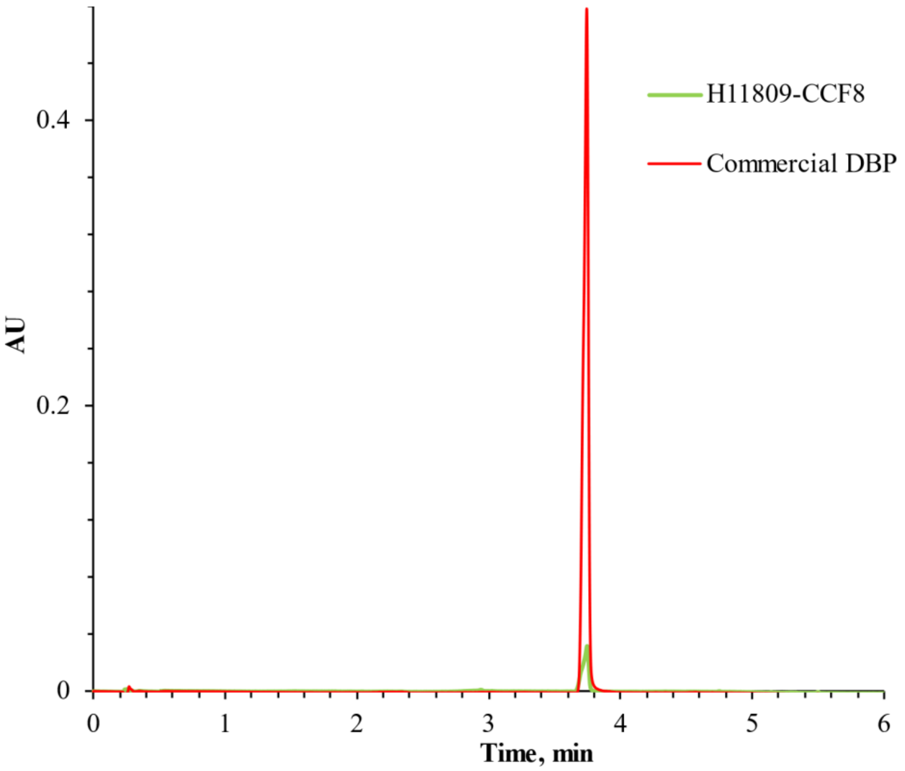

Figure S4 UPLC chromatogram of H11809-CCF8 (green) and commercial DBP (red), 5 µL (10 µg/mL) of working solution injected into UPLC, and the chromatogram was viewed at 254 nm. Both compounds have similar RT (~3.7 min), hence confirming the presence of DBP in H11809-CCF8.

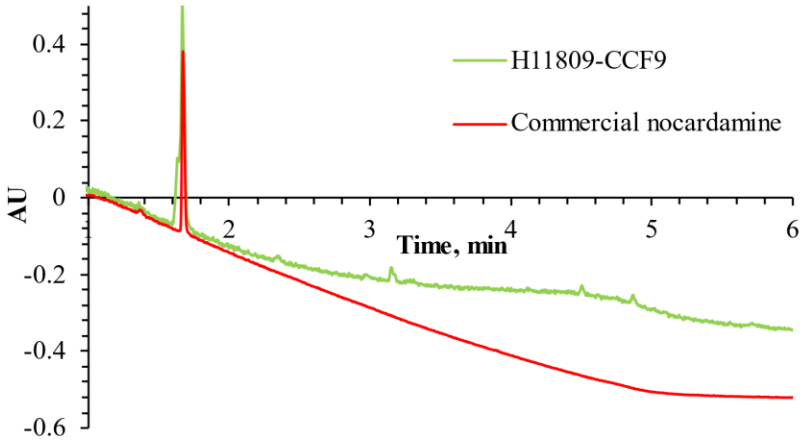

Figure S5. UPLC chromatogram of H11809-CCF9 (green) and commercial nocardamine (red), 5 µL (10 µg/mL) of working solution injected into UPLC, and the chromatogram was viewed at 200 nm. Both compounds have similar RT (~1.6 min), hence confirmed the presence of nocardamine in H11809-CCF9.

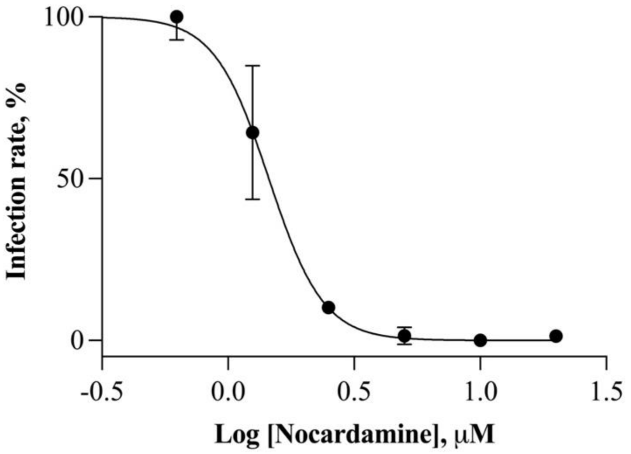

Figure S6. *Pf*3D7 IC_{50} determination of nocardamine (1.5 μM). The IC_{50} graph was plotted with GraphPad prism.

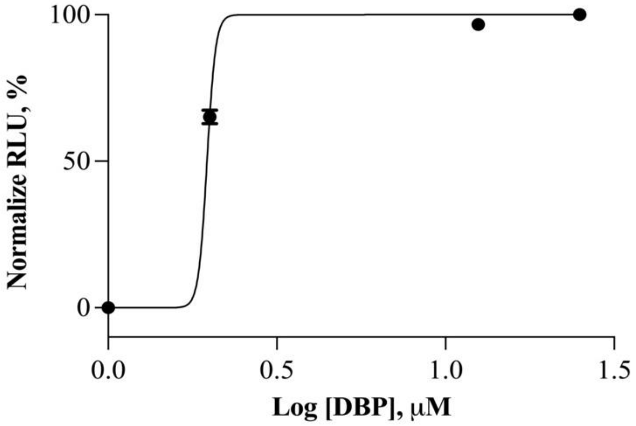

Figure S7. The IC_{50} determination of DBP against *Hs*GSK-3β. The IC_{50} of DBP against *Hs*GSK-3β was 2.0 μM, a moderate *Hs*GSK-3β inhibitor. The IC_{50} graph was plotted with GraphPad prism.

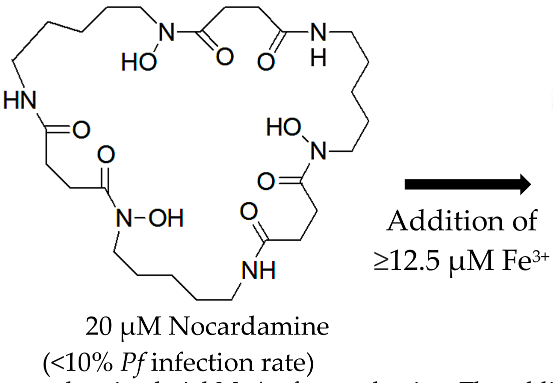

Figure S8. Suggested antimalarial MoA of nocardamine. The addition of ≥12.5 µM Fe^{3+} reduced the efficiency of nocardamine by 90% against *Pf* 3D7, indicating *Pf* iron starvation as the antimalarial MoA of nocardamine.

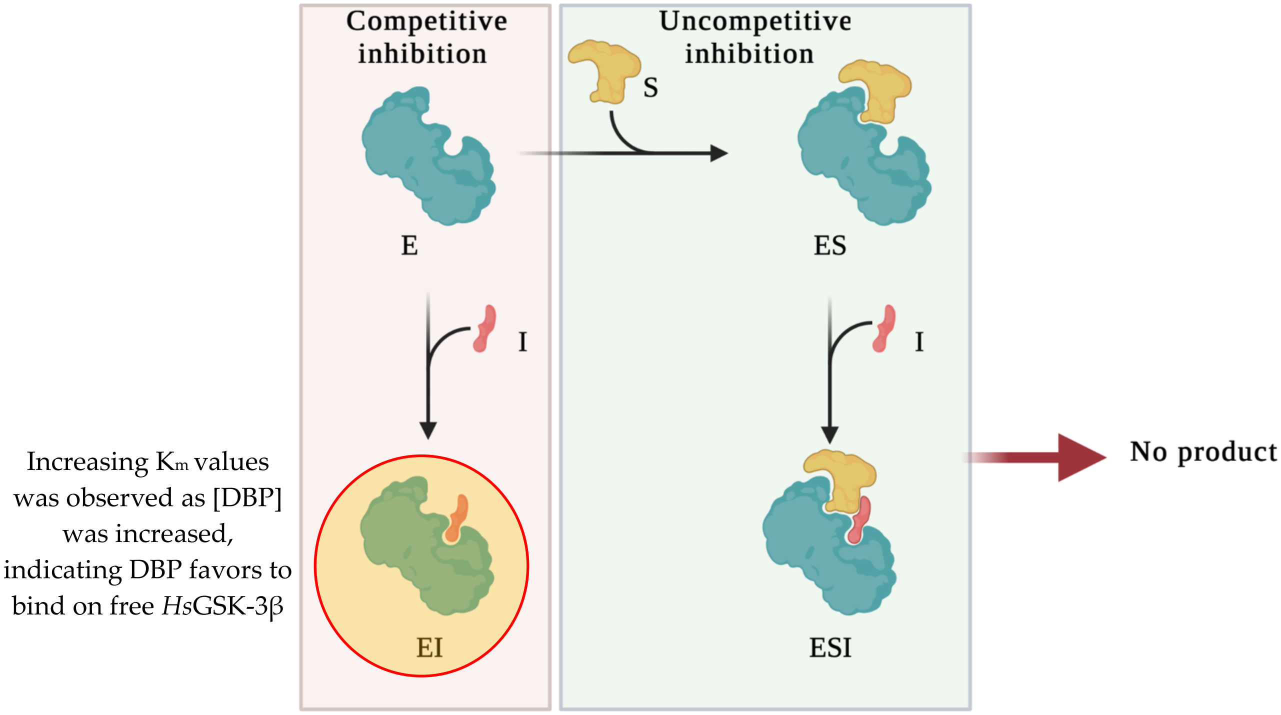

Figure S9. *Hs*GSK-3β inhibition MoA by DBP (I), postulated based on K_m and V_{max} changes. DBP was indicated to inhibit *Hs*GSK-3β via a mixed inhibition (a combination of competitive and uncompetitive inhibitions patterns). However, the increase of K_m values indicates that DBP favors/higher affinity to bind on free *Hs*GSK-3β/enzyme (E) than enzyme-substrate complexes (ES). E = Enzyme, S = Substrate, I = Inhibitor, ES = Enzyme-Substrate complex, EI = Enzyme-Inhibitor complex, ESI = Enzyme-Substrate-Inhibitor complex. Created with BioRender.com.
